# Digital technology integration in home-based exercise: a systematic review of research evolution, applications, and impact mechanisms

**DOI:** 10.1186/s12889-025-24679-9

**Published:** 2025-10-17

**Authors:** Tong Zhou, Shunan Zhang, Siqi Liu, Jieun Yu

**Affiliations:** 1 Department of Physical Education, Korea University, Seoul, 02841 Republic of Korea; 2 College of Chinese Language and Culture, Huaqiao University, Xiamen, 361021 China; 3 Faculty of Sports and Exercise Science, University Malaya, Kuala Lumpur, 50603 Malaysia

**Keywords:** Digital technology, Home-based exercise, Systematic review, Bibliometric, Digital health, Public health intervention

## Abstract

The integration of digital technologies in home-based exercise (HBE) has emerged as a critical public health intervention, particularly following the COVID-19 pandemic. However, comprehensive understanding of how digital technologies influence HBE research evolution and practice remains limited. This systematic review aims to: (1) map the knowledge evolution of digital technology-supported HBE research, (2) analyze innovative applications and value propositions of digital technologies in HBE, (3) identify key determinants of HBE participation in the digital era, and (4) predict future development trends. Following PRISMA guidelines, we conducted a comprehensive search of the Web of Science database from 2000 to 2024, yielding 311 articles for bibliometric analysis using CiteSpace and VOSviewer. Results indicated that digital technology-supported HBE research has grown exponentially, peaking in 2022. We identified seven core research themes, including intelligent exercise training, AI-driven fall prevention, wearable device interventions, virtual reality rehabilitation, mobile health applications, cancer patient tracking systems, and remote diagnostics. These themes highlight innovative approaches to personalized training, remote monitoring, and immersive rehabilitation experiences, demonstrating significant value in enhancing exercise adherence and health outcomes. Key determinants of HBE participation were identified at personal (social support, professional guidance, self-efficacy) and technological (digital literacy, accessibility, user interface design, data privacy) levels. Digital technologies demonstrate substantial potential for enhancing HBE accessibility, improving adherence, and reducing public health burden. Future research should prioritize addressing digital equity and developing evidence-based implementation frameworks to ensure sustainable and inclusive digital health interventions.

## Introduction

Given the increasing importance of home-based exercise (HBE) in improving public health, it is crucial to understand its role in reducing the public health burden, a key indicator of healthcare costs. Research has shown that physical inactivity has directly cost the global healthcare system $53.8 billion since 2013 [17]. More alarmingly, the global health burden due to severe physical inactivity continues to rise [39], with a prevalence of 27.5% among adults attributed to physical inactivity in 2016 [29]. The consequences of this trend, if left unaddressed, are projected to be severe. It is estimated that if the global prevalence of physical inactivity does not significantly improve, nearly 500 million new cases of preventable non-communicable diseases (NCDs) and $520 billion in associated healthcare costs will be incurred between 2020 and 2030 [72]. These staggering figures underscore the urgency of increasing physical activity levels. In this context, home-based exercise, as an easily accessible form of fitness, demonstrates enormous potential. It not only effectively improves the health of the entire population but also significantly reduces healthcare costs.

With the continuous enhancement of health awareness and the rapid development of technology, HBE is increasingly becoming a popular fitness approach. In recent years, major events such as the COVID-19 pandemic have further accelerated the popularization and advancement of HBE [37]. Compared to traditional gym workouts, HBE offers advantages such as greater convenience, increased time flexibility, and lower economic costs [18]. Specifically, individuals can engage in exercises at home utilizing various equipment or relying solely on bodyweight exercises based on their personal needs and schedules [30]. In addition to the traditional advantages, modern technology has brought new advantages to HBE, with real-time monitoring by wearable devices [10], personalized exercise advice by AI algorithms [2], exercise workouts by AR/VR [60], remote system guidance [90], and the rise of emerging media communication technologies and short-form video platforms providing a wealth of instructional resources for home health education [42] that meet the needs and requirements, especially for rehabilitation exercises for the elderly.

While the popularity of HBE continues to grow, there remains a critical need for in-depth exploration of the mechanisms of driving participation and the long-term effects of this fitness approach. Research indicates that a complex interplay of factors, including personal motivation, home environment, social support, and injury concerns, may significantly influence HBE engagement [31, 84]. A systematic review revealed that for patients sustaining exercise-related injuries, factors influencing their adherence to HBE primarily include social support, guidance, exercise frequency, self-motivation, self-efficacy, past adherence behavior, baseline physical activity or aerobic capacity level, exercise attentiveness, exacerbation of pain during exercise, and high levels of helplessness, depression, and anxiety [7]. The integration of digital technologies has introduced new dimensions to these factors, with smart devices providing real-time feedback, AI-powered virtual coaching offering personalized guidance, and wearable sensors enabling precise movement monitoring and injury prevention. Digital platforms also facilitate social connectivity and motivation through virtual communities and gamification elements. In addition, increased public health awareness [67] and the promotion of home exercise prescription [62] provide new contexts for research. Furthermore, the advancement of Internet of Things (IoT) devices and artificial intelligence algorithms has enabled more sophisticated tracking of exercise adherence patterns and health outcomes [28, 40]. By delving deeper into these complex interactions, a better understanding and promotion of home exercise participation is provided to improve overall population health.

The necessity for comprehensive trend analysis in home-based exercise research stems from several converging factors that have fundamentally transformed the landscape of physical activity and public health interventions. First, the demographic transition toward an aging global population has created unprecedented demand for accessible, cost-effective exercise solutions. Traditional facility-based exercise programs often present barriers including transportation difficulties, scheduling constraints, and financial limitations, particularly for elderly populations and those with chronic conditions [50]. Understanding trends in HBE research becomes critical for developing evidence-based strategies that can address these accessibility challenges while maintaining exercise effectiveness. Second, the rapid proliferation of digital health technologies has created a paradigm shift in exercise delivery and monitoring capabilities. From basic pedometers to sophisticated Ai-powered virtual coaches, the technological landscape has evolved dramatically since 2000. However, this technological advancement has outpaced our understanding of how these tools can be optimally integrated into home-based exercise programs. Trend analysis helps identify which technological innovations have demonstrated sustainable impact versus those that represent mere technological novelty. Third, the COVID-19 pandemic has accelerated the adoption of home-based exercise as a primary rather than supplementary form of physical activity. This shift has generated new research questions about long-term adherence, effectiveness, and scalability of home-based interventions. Understanding pre-pandemic trends versus pandemic-era developments provides crucial insights for post-pandemic exercise prescription and policy development. Finally, the growing recognition of physical inactivity as a global pandemic, with economic costs exceeding $53.8 billion annually, necessitates systematic understanding of how home-based exercise research has evolved to address this challenge. Trend analysis enables identification of research gaps, successful intervention strategies, and areas requiring future investigation to maximize public health impact.

Therefore, this study addresses four critical research questions that directly correspond to the analytical framework and expected outcomes:

- RQ1: Knowledge Evolution Mapping - How has digital technology-supported HBE research evolved from 2000 to 2024, and what are the key developmental phases and turning points?
- RQ2: Digital Technology Applications and Value - What are the primary digital technology applications in HBE research, and what evidence exists for their effectiveness and impact?
- RQ3: Participation Determinants and Mechanisms - What factors influence HBE participation in the digital era, and how do these factors interact to shape engagement patterns?
- RQ4: Future Development Prediction - Based on current trends and technological developments, what are the predicted future directions and innovation pathways for digital HBE research?

These research questions are designed to provide comprehensive insights into the current state, applications, mechanisms, and future directions of digital technology-supported home-based exercise research.

## Literature review

### Home-based exercise

Home-based exercise (HBE) refers to a variety of physical activities carried out within the domestic or family environment. Specifically, it involves transforming the household space into an exercise arena, where individuals can engage in a series of physical training activities independently or with family members, either utilizing bodyweight exercises or portable equipment. In this study, the concept of HBE was specifically defined to fit the society.

#### Characteristics of home-based exercise

##### The rapid popularity of HBE primarily stems from two factors

Firstly, the accelerated pace of life and increased work pressure in society have made it difficult for people to exercise regularly in places such as gyms. The convenience and flexibility of HBE effectively address this issue. It is not restricted by venue and can be performed at home, allowing individuals to exercise flexibly according to their own schedules, thus reducing time constraints [18].

Secondly, increased public health awareness has facilitated the recognition of the importance of sustained exercise, driving the adoption of low-cost, personalized home exercise programs. The rapid development of the Internet and communication technologies has been a significant driver in the rise of HBE [89]. Online fitness programs and instructional videos have enriched the form and content of home-based exercise. In particular, during the COVID-19 epidemic, online fitness instructional videos and classes contributed to the emergence of home exercise as an important way to maintain health, and its popularity was further fueled by a deepening sense of health awareness [45]. A variety of VR fitness games for HBE have also fueled a continued increase in public participation in home fitness [59, 91].

As articulated by Nyenhuis et al. [53], HBE encompasses a variety of forms. These include aerobic exercises such as jumping rope, brisk walking, and jogging for cardiovascular strengthening. On the other hand, a combination of aerobic, anaerobic, and strength training utilizes home equipment or bodyweight exercises like squats and sit-ups to develop muscle strength. Moreover, activities such as aerobic dance and tai chi can increase flexibility and relax the mind [44]. Furthermore, with the rise of internet fitness apps, online fitness programs have emerged, allowing people to create flexible combinations at home based on their personal goals and circumstances [3].

#### Current state of research on home-based exercise

HBE is diverse and interdisciplinary, with a major focus on outcome evaluation, analysis of participation factors, innovations in technology application and special population interventions [11, 49]. Different research methodological designs and HBE interventions provide a multifaceted perspective for a comprehensive understanding of HBE [27, 30] and tend to favor rehabilitation mostly, covering different populations such as older adults, women and adolescents [80, 93]. Research has confirmed the positive effects of HBE on improving physical health, mental health and quality of life [1]. However, HBE faces some challenges in terms of long-term adherence compared to traditional gym-based exercise.

#### Emerging trends and technology applications in home-based exercise

Technological advancements have brought new opportunities for HBE. Studies have found that wearable devices, smartphone apps, virtual and augmented reality, and online fitness communities can be used to instruct, and monitor HBE [4, 74]. Regarding the HBE needs of special populations, some studies have focused on the requirements of specific age groups or those with particular health conditions, such as children, the elderly, individuals with disabilities, and pregnant women [19, 32, 76]. The content of HBE interventions varies for different populations. Additionally, the rapid pace of technological change often outpaces rigorous evaluation of long-term effectiveness and safety, creating gaps between technological capability and evidence-based implementation.

Despite the growing of research on digital technology-supported HBE, there remains a lack of systematic understanding of research evolution, interdisciplinary connections, and future research priorities. Specifically, there is limited understanding of how digital technologies have evolved within HBE research, how interdisciplinary knowledge networks have developed, and which technological innovations have demonstrated sustained research interest and practical application. This study addresses these gaps by employing bibliometric analysis to comprehensively map the research landscape of digital technology-supported HBE.

### Bibliometrics analysis

Bibliometric analysis serves as a particularly appropriate methodological approach for understanding the evolution of digital technology-supported home-based exercise research for several strategic reasons directly related to the study’s objectives. Bibliometrics employs clustering algorithms and normalization algorithms to reveal trends, topics, and keyword information within a research domain [21]. By analyzing citations, authors, journals, and other bibliographic information, it helps researchers understand the developmental trajectories and academic contributions in a given field [92]. Unlike meta-analyses, bibliometrics does not need to consider the potential adverse effects of heterogeneity and publication bias in existing studies [38]. This method enables objective and quantitative analysis of large-scale literature, providing insights into the evolution and current state of digital technology applications in HBE. Specifically, CiteSpace and VOSviewer were used to identify research trajectories, interdisciplinary connections, and influential studies, facilitating a comprehensive understanding of the research domain.

## Method

This review is based on the PRISMA guidelines. And this systematic review protocol has been registered on INPLASY (inplasy.com). The registration number is INPLASY202470018(DOI:10.37766/inplasy2024.7.0018). This protocol was performed in accordance with the preferred reporting items for systematic reviews and meta-analysis protocol.

### Literature screening criteria

To include all relevant “home-based exercise” articles in this study, each study had to meet the criteria in Table 1.

**Table 1:** Inclusion and exclusion criteria

Inclusion	Exclusion
a. All studies related to home-based exercise	a. Conference papers and book chapters are not included
b. Search period from early 2000 to 1 June 2024	b. Languages in English
c. Indexed in SCI, SSCI, A&HCI	
d. Peer review	
e. Technology-related articles in HBE	

### Data sources and search strategies

This scoping literature review identified relevant studies through systematic searches of publicly available literature. Following the research objectives, we conducted searches within the Web of Science (WoS) core database [65]. As a comprehensive platform containing high-quality peer-reviewed literature, WoS ensures the reliability and credibility of included articles.

The specific search strategy employed was: TS= ((“Home-based exercise”) OR (“Home-based training”) OR (“Home exercise program”) OR (“Home-based physical activity”) OR (“Home-based physical exercise”) OR (“Home-based physical program”) OR (“Home-based exercise program”)). The search encompassed the period from January 1, 2000, to June 1, 2024. After applying filters for SCI, SSCI, and A&HCI indexes, 3,014 results were retrieved. Subsequently, document types were restricted to articles only, excluding conference papers and book chapters, yielding 2,341 articles. Due to language proficiency constraints of the review team, studies Published primarily in English were selected, resulting in 2,311 articles. Following the exclusion of seven manifestly irrelevant items, a comprehensive database of 2,304 articles was established for overall bibliometric analysis.

The selection of the 2000–2024 timeframe represents a strategically significant period in digital health evolution. The year 2000 marked the post-dot-com bubble stabilization of digital technologies and the emergence of Web 2.0 paradigms, which fundamentally transformed healthcare delivery mechanisms [24, 55]. Concurrently, this period witnessed the establishment of regulatory frameworks for digital health devices by agencies such as the FDA, providing institutional support for the standardization of home-based exercise technologies. The endpoint of June 1, 2024, ensures comprehensive coverage of rapid technological developments and innovations in home-based exercise interventions during and following the COVID-19 pandemic.

**Fig. 1:**
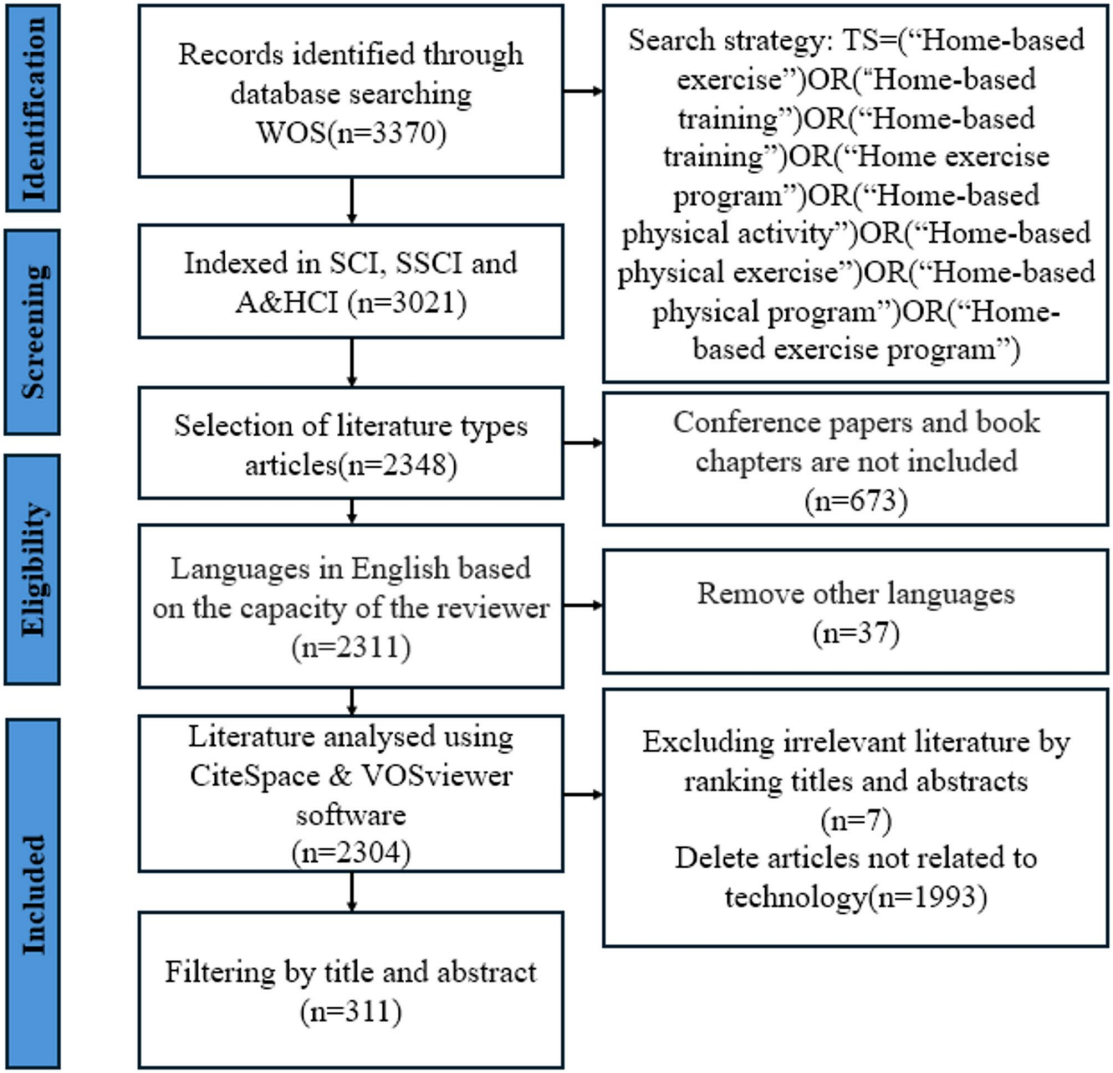
PRISMA flow chart

To minimize omissions and further validate technology-related research domains within HBE, two researchers independently assessed inclusion and exclusion criteria for each study based on titles and abstracts. Discrepancies were resolved through discussion with a third reviewer, achieving an inter-rater reliability of *R* = 99.95%. Through this rigorous secondary screening process, studies specifically addressing technology applications in home-based exercise were identified from the comprehensive database of 2,304 articles. Ultimately, 311 articles met the stringent inclusion criteria and were incorporated into the in-depth analysis. The detailed literature screening process is illustrated in Fig. 1.

This study employs a two-stage analytical approach: first, comprehensive bibliometric analysis was conducted on the complete dataset of 2,304 articles to elucidate overall developmental trends and knowledge structures in home-based exercise research; second, in-depth content analysis was performed on the 311 technology-focused articles to examine specific applications and developmental trajectories of technology in home-based exercise interventions.

### Data analysis

This study employs two visualization software tools, CiteSpace and VOSviewer, for bibliometric analysis to gain comprehensive and in-depth research insights.

CiteSpace excels at revealing evolutionary trends and knowledge structures in research fields [13]. In contrast, VOSviewer specializes in displaying the overall knowledge landscape of the research field [85]. CiteSpace provides a longitudinal perspective on temporal evolution, while VOSviewer offers a lateral view of the knowledge network. By combining these tools, we can gain a more comprehensive and in-depth understanding of the development history, topic distribution, and knowledge structure of HBE research.

## Results

### HBE trajectory, including keywords, authors, countries

#### Presentation of publication trend in home-based exercise

In this study, we analyzed the development of HBE using both CiteSpace and VOSviewer software. The article data collected from the Web of Science (WOS) shows that we gathered a total of 2,304 relevant Publications from 703 journals, 12,048 authors, 3,413 research institutions, and 73 countries. We analyzed the Publication quantity information, keywords, major research countries, and timeline of development for HBE. Additionally, these Publications produced a total of 6,667 keywords and analyzed the strength and burst information of these keywords.

Publication output and growth trend, as well as the number of peer-reviewed publications, are important indicators for evaluating the development trend of a research discipline or field.

**Fig. 2:**
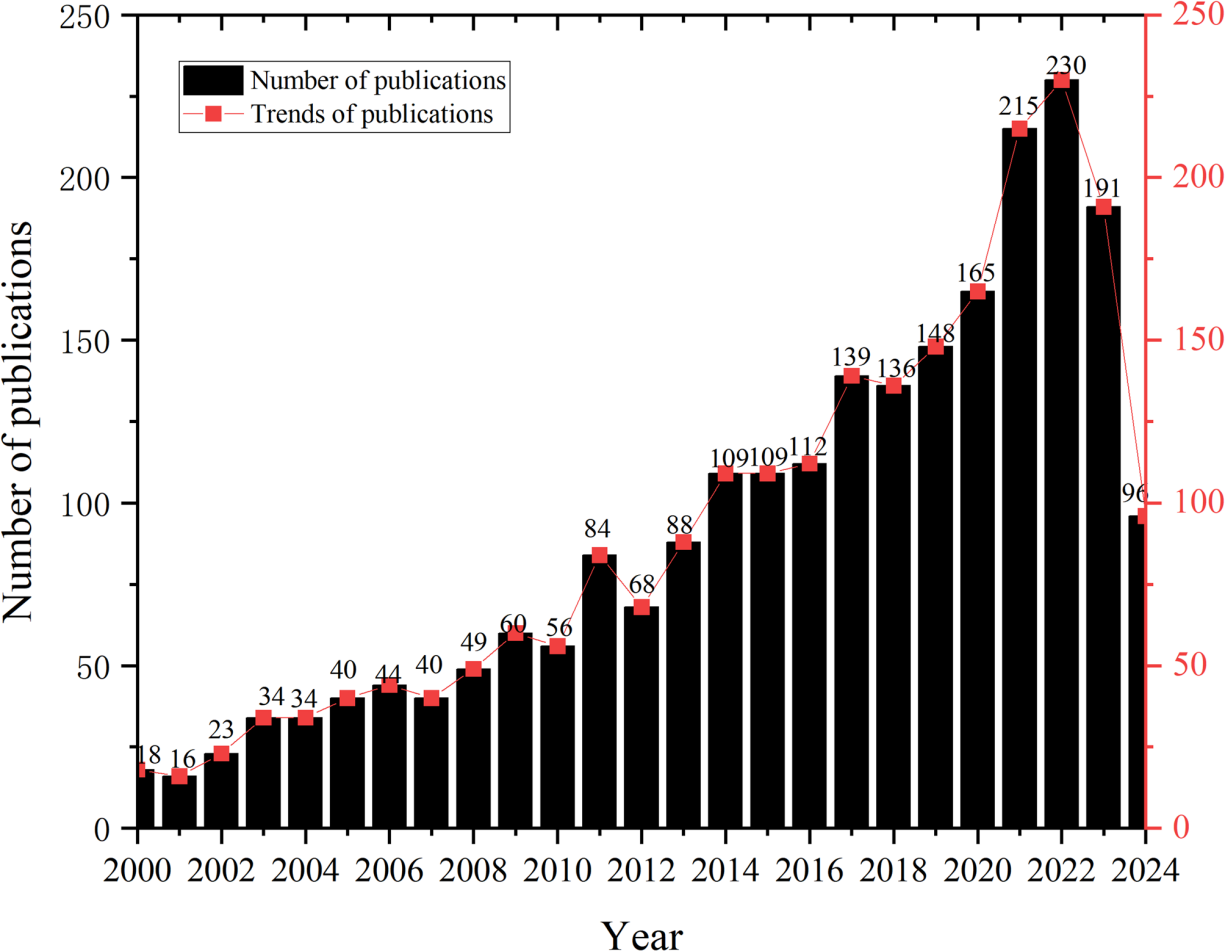
Publications in home-based exercise from 2000 to 2024

As shown in Fig. 2, starting from 2000 (*n* = 18), the quantity of HBE Publications has shown a continuous upward trend, reaching its peak in 2022 (*n* = 230), followed by 2023 (*n* = 191) and up to June 2024 (*n* = 96). This phenomenon can be explained by Price’s law [66], where a research field begins with a small group of scientists publishing articles, followed by exponential growth, then consolidation of the knowledge system and a decrease in publication quantity, and finally, a mature inflection point is reflected. Due to the COVID-19 pandemic, many articles focused on HBE to promote physical activity [27] during this period, leading to the exponential increase.

#### Major authors and countries in home-based exercise

**Fig. 3:**
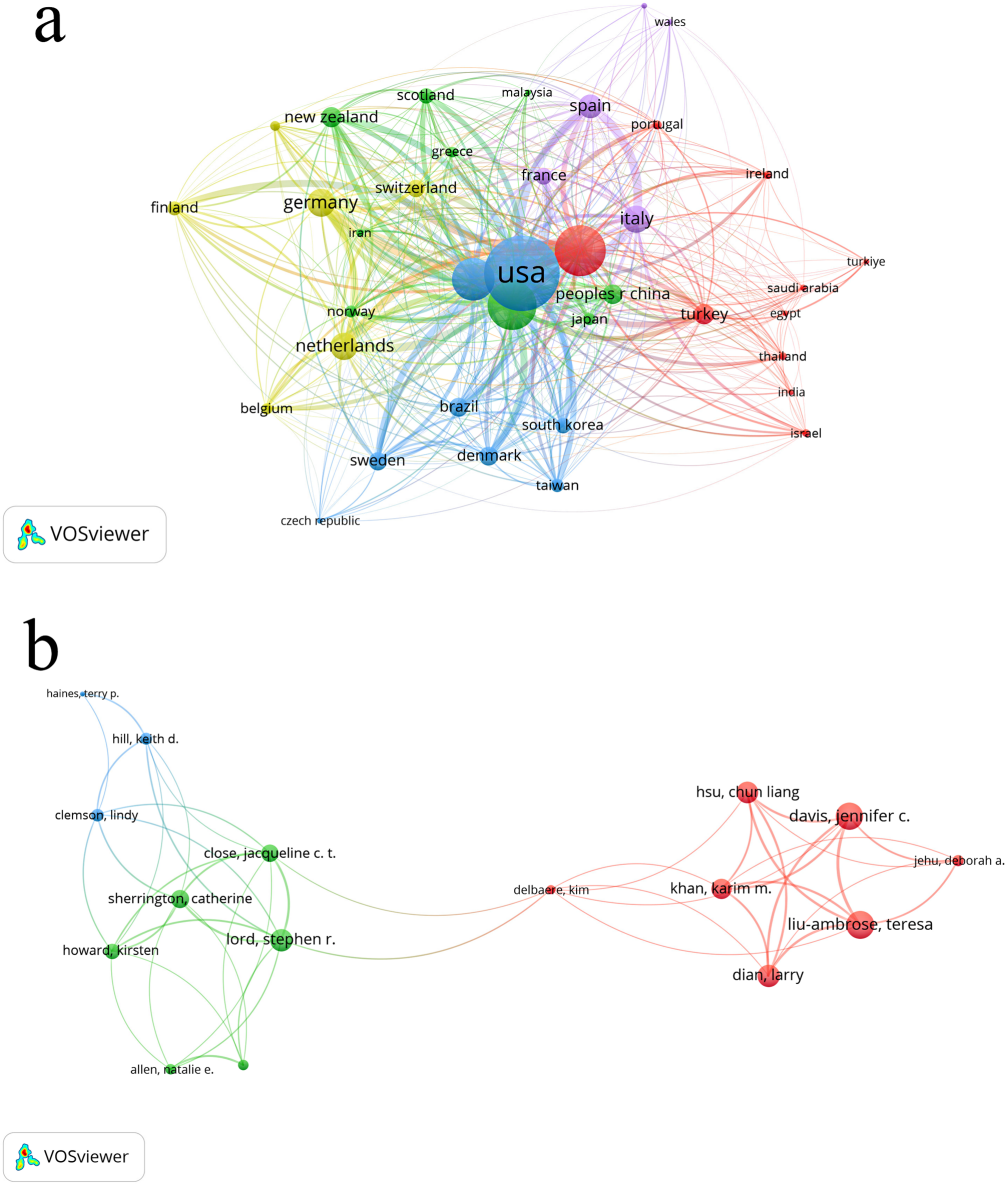
**a** Productive authors publishing on home-based exercise. **b** Productive countries publishing on home-based exercise. Image source: Author’s own analysis using the VOSviewer software

Figure 3(a) shows the authors who have published articles on the topic of HBE, and Table 2 presents the top 5 most productive authors. The ranking of prolific authors is based on total Publication output within the analyzed corpus. Kautiainen, Hannu emerges as the most productive author, contributing 17 articles (representing 0.70% of the total corpus, with an average citation rate of 32 citations per article). Hill, Keith D. follows with 16 publications (0.69%, averaging 24.875 citations per article), succeeded by Manfredini, Fabio with 15 articles (0.65%, averaging 14.8 citations per article). Lamberti, Nicola and Lord, Stephen R. each contributed 14 articles (0.61% respectively), though their average citation impacts differ substantially at 12.071 and 104.571 citations per article, respectively.

**Table 2:** Top-5 of most productive authors publishing on home-based exercise

No.	Author	Documents	Citations	Total link strength	Average citations
1	Kautiainen.Hannu	17	544	37	32
2	Hill. Keith D.	16	398	12	24.875
3	Manfredini. Fabio	15	222	49	14.8
4	Lamberti. Nicola	14	169	49	12.071
5	Lord. Stephen R.	14	1464	27	104.571

While Lord, Stephen R.‘s publication volume (14 articles) does not rank among the top three, his average citation impact of 104.571 citations per article significantly exceeds that of other authors, indicating exceptional research influence and quality. This metric (parenthetical figures denote average citations per article) demonstrates the divergent relationship between research productivity and scholarly impact, wherein publication quantity does not invariably correlate with research quality or influence.

Figure 3(b) presents the major countries contributing to HBE research, as shown in Table 3 with the top 10 most productive countries. The United States Published the highest number of articles with 650 (28.21%, 39.823) and received 25,885 citations, making it the most productive country. Australia follows with 270 (11.72%, 37.389) articles, and England ranks third with 203 (8.81%, 43.660) articles. Canada has the highest average citations per paper, with 192 articles (8.33%, 52.625). These findings reflect the emphasis placed on physical activity, health, and HBE by these countries, as well as their academic strength in this field.

**Table 3:** Top-10 of most productive countries publishing on home-based exercise

No.	Country	Documents	Citations	Total link strength	Average citations
1	USA	650	25,885	243	39.823
2	Australia	270	10,095	196	37.389
3	England	203	8863	200	43.660
4	Canada	192	10,104	129	52.625
5	Turkey	170	3091	21	18.182
6	Germany	127	2689	139	21.173
7	Italy	110	3283	101	29.845
8	Netherlands	101	3694	100	36.574
9	China	99	1234	37	12.465
10	Denmark	79	1888	67	23.899

#### Keyword analysis for home-based exercise

To comprehensively understand the research landscape of home-based exercise (HBE), it is essential to analyze the centrality and specific occurrences of keywords. This can be achieved by examining the network metrics and visualizations of these keywords using tools like CiteSpace. The centrality of keywords provides insights into their importance within the research domain, while their occurrences highlight prevalent themes.

**Fig. 4:**
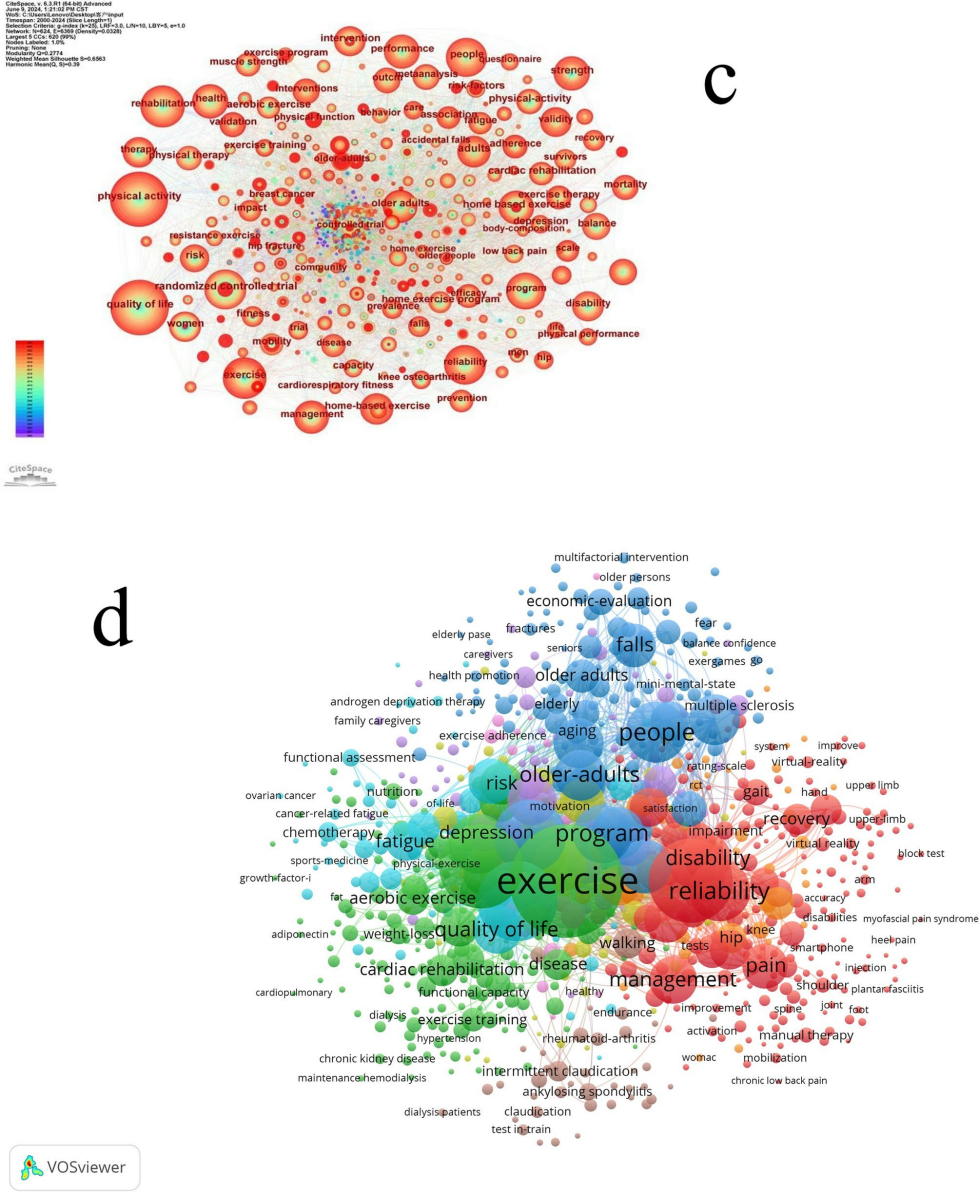
c. Keyword co-occurrence for home-based exercise using CiteSpace; d. Keyword co-occurrence for home-based exercise using VOSviewer. Image source: Author’s own analysis using the software

In Fig. 4, the keyword network generated by CiteSpace reveals the interconnections between various terms, with denser clusters indicating more closely related research themes. For instance, keywords such as “physical activity,” “home-based exercise,” and “cardiac rehabilitation” form a central cluster, suggesting that these topics are frequently studied together. The network’s density and modularity can further inform us about the cohesion and diversity of research areas within HBE.

#### Bursting keywords in home-based exercise

In CiteSpace, a “burst term” refers to a phenomenon where the frequency or citation count of a word suddenly and dramatically increases within a specific time period. In CiteSpace, a “burst term” refers to a phenomenon where the frequency or citation count of a word suddenly and dramatically increases within a specific time period. This indicates a surge in research interest and activity around that particular term.

**Fig. 5:**
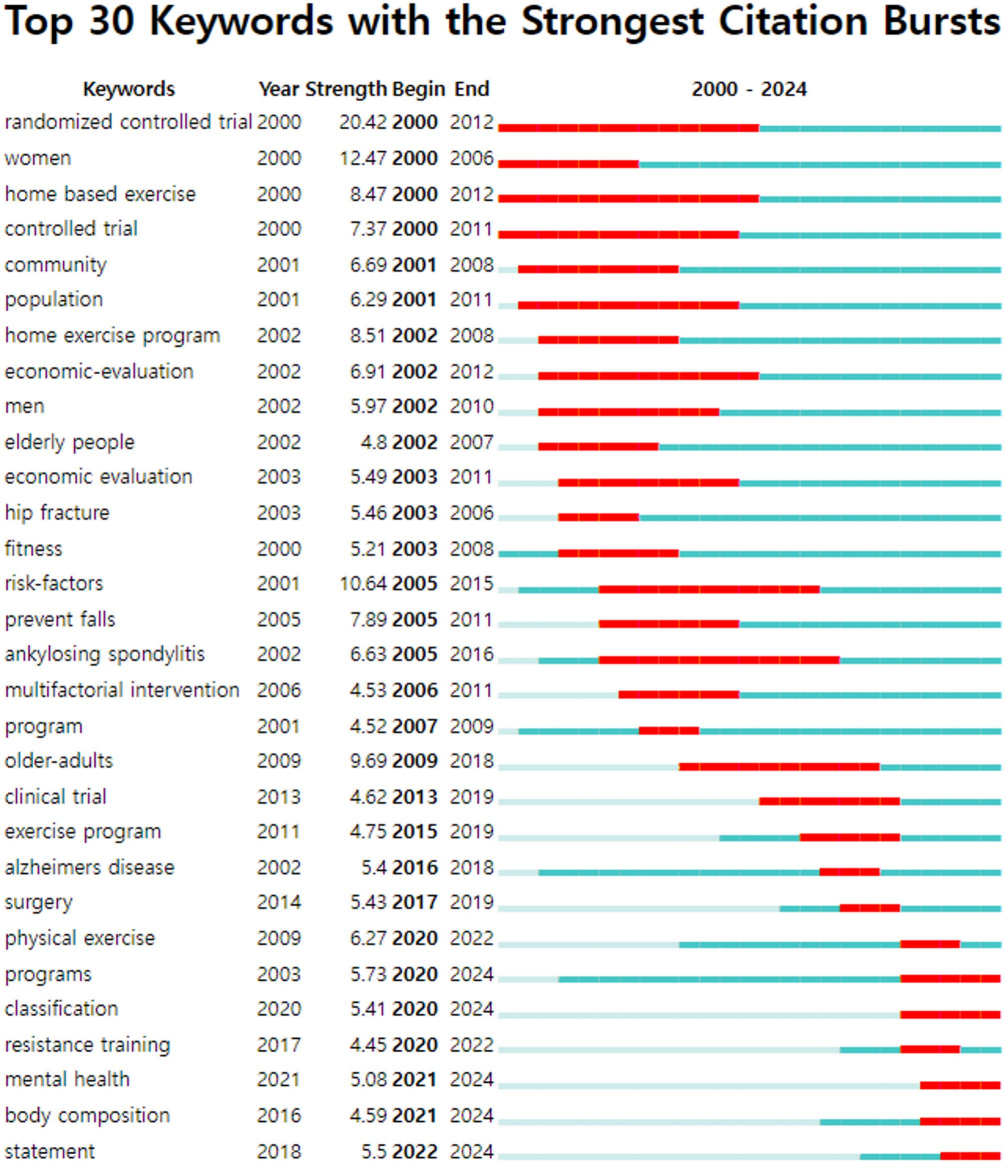
Top 30 keywords with the strongest citation burst term on home-based exercise

Figure 5 displays the 30 major bursting keywords related to HBE. The analysis revealed that “randomized controlled trial” had the highest burst strength of 20.42, as a burst term from 2000 to 2012, spanning 12 years. Table 4 shows that “women” (12.47, 2000–2006) and “risk factors” (10.64, 2005–2015) had the next highest burst strengths, closely followed by “older adults” (9.69, 2009–2018). Additionally, the analysis discovered that “programs” (5.73, 2020–2024), “mental health” (5.08, 2021–2024) and “body composition” (4.59, 2021–2024) are bursting terms that continued to appear until 2024, indicating their potential for further investigation.

### Research hotspots in home-based exercise

CiteSpace offers two powerful visualization tools for analyzing the distribution and evolution of keywords in HBE research: clustering analysis. The specific keywords for each cluster are detailed in Table 4.

**Table 4:** 7 keywords clusters of home-based exercise

keyword’s clusters/Theme	Keywords
#0 Intelligent exercise training	physical activity; home-based exercise; colorectal cancer; psychological health; blood pressure; cardiac rehabilitation; hospital-based exercise; arterial stiffness; cardiac allograft; social perceptions
#1 Falls risk prevention driven by AI	physical activity; multiple sclerosis; exercise adherence; targeted behavior change intervention; lower extremity function; home based exercise; randomized controlled trial; balance; older persons; adults
#2 Wearable devices and digital musculoskeletal disease interventions	exercise therapy; spinal fusion; surgical decompression; guillain-barre syndrome; neck cancer; knee osteoarthritis; therapeutic ultrasound; bodyweight-based exercise training; musculoskeletal disorder; joint mobility
#3 virtual reality technology for neurological disease rehabilitation	multiple sclerosis; physical therapy; mesenchymal stem cells; smartphone; sacroiliac joint; home-based training; manual wheelchair; cognitive computer games; primary health care; bladder neoplasm
#4 MHealth solutions for mental and cardiovascular health testing	physical activity; exercise program; cardiac risk factors; hybrid models; regular physical activity; home-based exercise; lung cancer; exercise capacity; vascular medicine; smartphone
#5 Rehabilitation tracking systems for cancer patients	physical activity; home-based training; red blood cells; adjuvant chemotherapy; chemotherapy-induced peripheral neuropathy; breast cancer; acute leukemia; systematic review; acute leukemia survivors; patient-reported symptoms
#6 Remote web-based diagnostics and rehabilitation of peripheral vascular disease	diagnosis; survivors; adjustment; quality of life; questionnaire; amyotrophic lateral sclerosis; fatigue; clinical trials; mouse model; scale

From Table 4 each cluster represents a distinct research hotspot in HBE, highlighting areas where significant advancements and innovations are being made. For instance, Cluster #0 focuses on intelligent exercise training, integrating technology with traditional exercise programs to enhance outcomes for various health conditions, such as colorectal cancer and cardiac rehabilitation. Cluster #1 emphasizes AI-driven fall risk prevention, particularly relevant for older adults and those with mobility issues.

Our analysis provides the prevalence of each factor, key research findings, and representative studies, while also highlighting seven major technology implementation areas in HBE: intelligent exercise training and remote rehabilitation coaching, AI-driven fall risk prevention, wearable devices and digital musculoskeletal disease interventions, virtual reality technology for neurological disease rehabilitation, mHealth solutions for mental and cardiovascular health testing, rehabilitation tracking systems for cancer patients, and remote web-based diagnostics and rehabilitation of peripheral vascular disease.

**Fig. 6:**
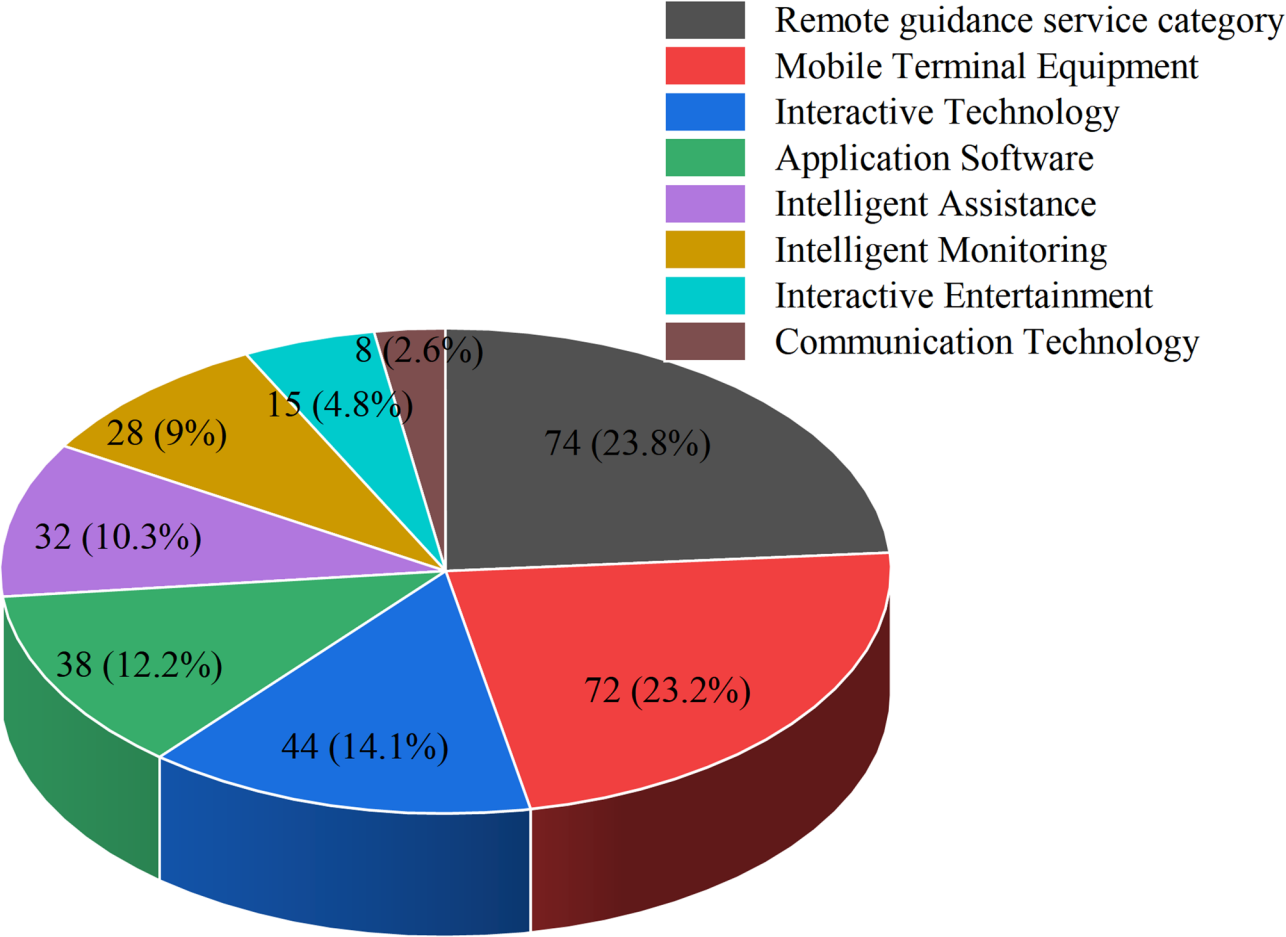
Type of technology in HBE

Here is a comprehensive overview of the eight technological categories identified from the 311 articles on HBE applications in Fig. 6. (1) Mobile Terminal Devices(72, 23.2%), including smartphones, tablets, portable devices, wearable devices (such as pedometers and accelerometers), and mobile devices; (2) Interactive Technologies(44, 14.1%), comprising virtual reality technology, visual feedback technology, gamification technology, and 3D technology; (3) Intelligent Monitoring(28, 9%), consisting of sensor technology, remote monitoring systems, remote detection technology, telephone supervision system, remote surveillance technology, and accelerometer sensors; (4) Application Software(38, 12.2%), including mobile applications, exercise applications, computer programs, online platforms, digital platforms, and e-health systems; (5) Communication Technologies(8, 2.6%), encompassing remote communication technology, video conference technology, online network technology, and remote live streaming technology; (6) Intelligent Assistance(32, 12.2%), featuring robotic assistance, computer assistance, voice assistants, digital voice assistants, and machine learning technology; (7) Interactive Entertainment(15, 4.8%) including video games, electronic games, exercise games, computer games, and gaming consoles; and (8) Remote Guidance Services(74, 24.8%), comprising remote rehabilitation guidance, online exercise programs, remote telephone guidance, online training, and interactive remote assistance.

**Fig. 7:**
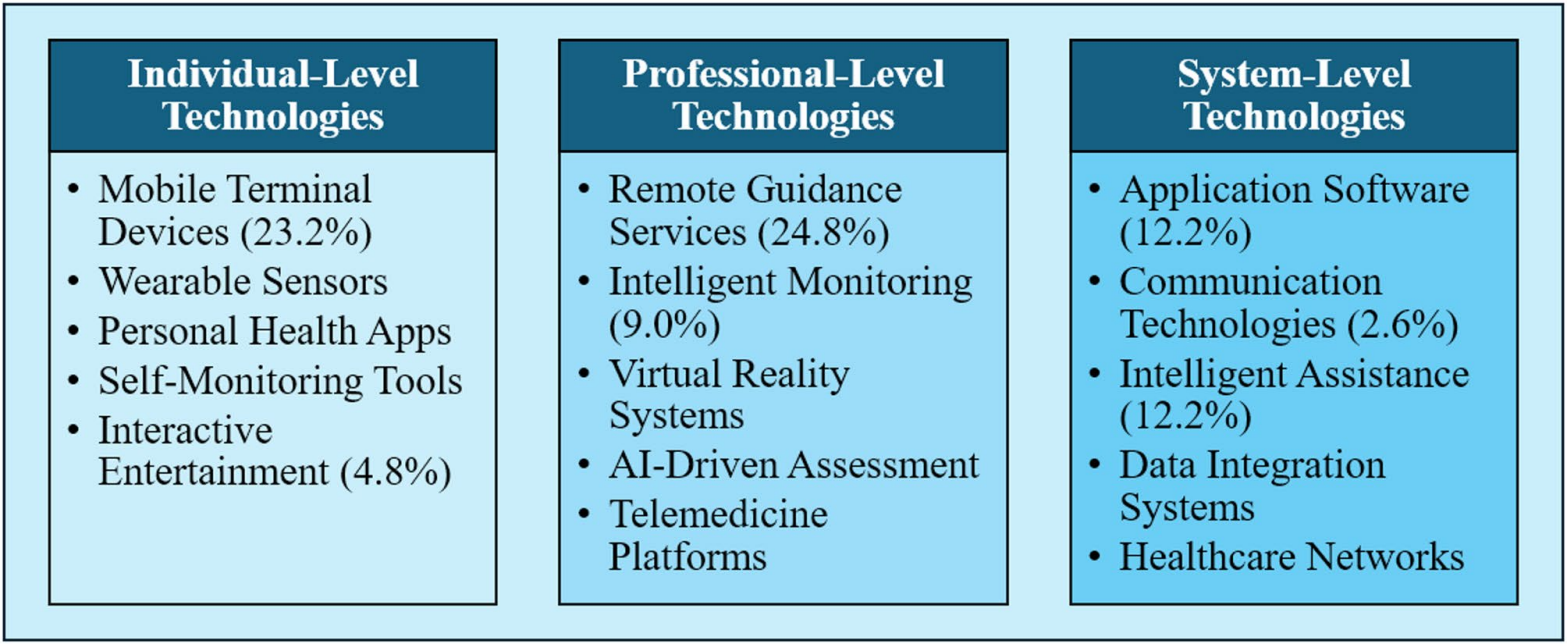
The HBE technology integration framework illustrates three distinct levels of technology integration: individual-level technologies focusing on personal engagement and monitoring, professional-level technologies enabling remote supervision and clinical guidance, and system-level technologies facilitating comprehensive health management

To better illustrate the complex relationships and evolutionary patterns identified in this study, we developed a comprehensive conceptual framework (Fig. 7) that visualizes the integration of seven technology categories with health outcomes in HBE. This framework demonstrates how different digital technologies (Mobile Terminal Devices, Interactive Technologies, Intelligent Monitoring, etc.) interconnect to support various health conditions and populations.

The framework reveals three distinct integration levels: (1) Individual-level technologies focusing on personal monitoring and engagement, (2) Professional-level technologies enabling remote supervision and guidance, and (3) System-level technologies facilitating comprehensive health management. (Percentages indicate the relative prevalence of each technology category based on analysis of 311 HBE application studies).

**Fig. 8:**
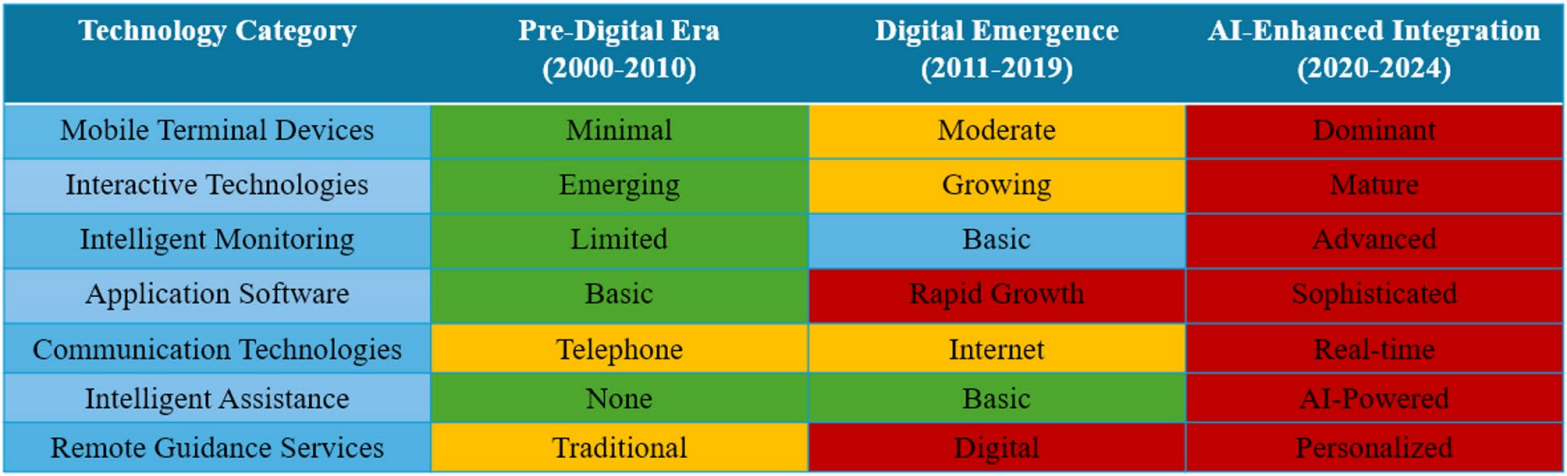
The temporal evolution matrix demonstrates the progression of HBE technologies across three distinct periods. Color coding indicates development intensity: green (low/emerging), orange (moderate/growing), and red (high/dominant)

Furthermore, we constructed a temporal evolution matrix (Fig. 8) showing the emergence and maturation of different technology categories from 2000 to 2024, highlighting critical transition periods including the pre-digital era (2000–2010), digital emergence (2011–2019), and AI-enhanced integration (2020–2024). This visualization clearly demonstrates how the COVID-19 pandemic served as a catalyst for accelerated technology adoption, with mobile terminal devices and remote guidance services showing the most significant growth during 2020–2022.

### Awareness and participation factors in home-based exercise

Through comprehensive literature analysis, we identified two categories of key factors influencing HBE perceptions and participation. The first category includes six traditional factors: social support, professional guidance, frequency of exercise, personal factors (e.g., self-efficacy), baseline fitness status, and lifestyle and emotional factors in Table 5. The second category encompasses digital technology factors that emerged from recent research: digital literacy, technology accessibility, user interface design, data privacy concerns, and virtual coaching support in Table 6.

**Table 5:** Awareness and participation factors in home-based exercise

Factor	Prevalence in Studies	Key Findings	Representative Study
Social Support	Emphasized as important	Emotional encouragement and practical assistance from family, friends, and peers can significantly improve patients’ adherence to HBE.	Jack et al. [35]
Professional Guidance	Considered to play a crucial role	Guidance, feedback, follow-up, and supplementary educational materials provided by physical therapists and other healthcare professionals can enhance patients’ exercise motivation and cultivate good exercise habits	Jordan et al. [36]
Exercise Frequency	Confirmed as important	Individuals aged 65 and above are more Likely to persist with an exercise frequency of 3 times per week or less; college students find adhering to about 3 times per week more sustainable	Eckard et al. [23, 70]
Personal Factors	Mentioned in multiple studies	Self-motivation levels, self-efficacy, and past adherence behaviors affect the degree of participation in HBE; groups with different levels of self-efficacy exhibit varying levels of participation	Marrero et al. [48, 62]
Baseline Physical Condition	Identified as an influential factor	Individuals with a good level of physical activity or aerobic capacity at baseline are more likely to adhere to a HBE program	Geraedts et al. [26]
Lifestyle and Emotional Factors	Noted as significant barriers	Lack of time for exercise, forgetfulness, exacerbation of pain during exercise, or negative emotions like depression, anxiety, and helplessness can become obstacles	Sandford et al. [71]

**Table 6:** The importance of technology in HBE

Factor	Key Considerations	Implementation Areas
Digital Literacy	User’s ability to engage with digital platforms and technologies	Intelligent exercise training, Remote rehabilitation coaching
Technology Accessibility	Access to required devices and internet connectivity	Wearable devices, mHealth solutions
User Interface Design	Ease of use and intuitive navigation of digital platforms	Virtual reality technology, Remote web-based systems
Data Privacy and Security	Protection of personal health information and exercise data	Rehabilitation tracking systems
Virtual Coaching Support	Quality and effectiveness of digital guidance systems	AI-driven fall prevention, Remote rehabilitation

## Discussion

This study systematically assessed the research trends in HBE from 2000 to 2024 through bibliometric analysis. It analyzes publication trends, keywords, authors, and countries related to HBE literature and makes divisions based on the categories of technologies used in HBE and categorizes research hotspots as well. Finally, the study also explores the factors influencing awareness and participation in HBE at both levels, traditional perspective and under digital technology.

### Development of home-based exercise

In terms of the development trend of HBE from 2000 to 2024, research in this area has shown exponential growth. However, the latest research trends indicate that the output in this field has reached an inflection point. The COVID-19 pandemic led to a peak in HBE research, as HBE can effectively help people maintain physical activity during major disasters [27]. In the years following COVID-19, it remains to be seen whether the declining trend in HBE will continue.

Ranked by the number of Published articles, the top 5 are Kautiainen Hannu, Hill Keith d., Manfredini Fabio, Lamberti Nicola, and Lord Stephen r., with Lord Stephen r. having the highest average number of citations.

Specifically, Kautiainen Hannu, as the author with the highest number of publications, his research should have focused on clinical trials and effect evaluations of HBE, particularly on rehabilitation interventions for the elderly population. HBE has shown effectiveness in improving physical function and activity ability in Alzheimer’s patients [63, 64].

According to the bibliometric analysis, Hill Keith D.‘s research should have emphasized topics such as fall prevention, balance training, and the design and implementation of HBE programs for the elderly, women, and high-risk patients [5, 33, 88]. Manfredini Fabio’s research interests might have focused on evaluating the impact of exercise rehabilitation on chronic disease patients (e.g., cardiovascular diseases, diabetes) and optimizing relevant home-based training programs [8, 47].

Based on the previous topic clustering analysis, Lamberti Nicola may have specialized in HBE rehabilitation research for peripheral artery disease and stroke patients [41, 46]. With the highest number of citations, Lord Stephen R. should be an authoritative scholar in this field, with a relatively broad research scope covering various aspects of HBE, such as fall risk assessment for the elderly, exercise intervention strategies, and physical function promotion [68, 78, 87].

Ranked by the number of publications, the United States ranks first, followed by Australia, the United Kingdom, Canada, and other developed countries. Canada has the highest average number of citations per article, reflecting the leading position of these countries in research on physical activity, health, and HBE.

### Research hotspots in home-based exercise

#### Through bibliometric analysis, the 6,667 keywords were clustered into 7 main topics, as follows

##### Intelligent exercise training and rehabilitation

HBE interventions have shown effectiveness in improving both physical and psychological health. Personalized HBE programs can enhance physical function [34], while also positively impacting physiological indicators like blood pressure [6]. These programs are particularly beneficial for rehabilitation in conditions such as colorectal cancer.

##### Fall risk prevention driven by AI

Focusing on the elderly population, this area encompasses fall risk assessment, lower limb function improvement, and balance training. Research indicates that supervised exercise is more effective than unsupervised exercise in reducing fall risks [20]. Notably, these interventions are beneficial not only for the elderly but also for adults [5].

##### Wearable devices and digital musculoskeletal disease interventions, virtual reality technology for neurological disease rehabilitation

HBE has proven effective in treating musculoskeletal disorders, including knee osteoarthritis and spinal conditions. Studies have shown that HBE interventions can significantly relieve osteoarthritis pain [3], demonstrating the practical effectiveness of these approaches. Fukaya et al. [25]’s study demonstrates the feasibility of tracking frailty trajectories during inpatient rehabilitation after direct cardiac surgery based on kinematic measurements extracted using a single wearable sensor.

##### Virtual reality technology for neurological disease rehabilitation

HBE with physiotherapy, stem cell therapy, and digital aids has shown promise in promoting motor and cognitive restoration [81]. Long-term HBE has also demonstrated potential in improving executive function in older adults with memory impairment [56]. Pekyavas and Ergun [61] found that using a virtual reality exercise game program was more effective in the short term than a home exercise program.

##### MHealth solutions for mental and cardiovascular health testing

HBE has shown positive effects on psychological health, depression, anxiety, and quality of life. In cardiovascular health, HBE programs have effectively improved exercise capacity in patients with heart failure [22]. Van Beek et al. [86] found that an app-based dexterity program will improve dexterity in the short and long term and can be effective in improving finger and hand function, which is expected to generalize to improved activities of daily living and quality of life.

##### Rehabilitation tracking systems for cancer patients

From pre-treatment physical activity to post-operative recovery, HBE using remote coaching and monitoring has become an important tool in cancer rehabilitation [54, 73]. It has shown particular promise in alleviating chemotherapy side effects and as a long-term rehabilitation option for breast cancer patients [58].

##### Remote web-based diagnostics and rehabilitation of peripheral vascular disease

This involves diagnostic methods for peripheral artery disease and related diseases, as well as the assessment of exercise fatigue and other relevant indicators to optimize rehabilitation programs [9, 16, 41]. Lamberti et al. [41], in a 10-year retrospective study, found that active participation in HBE programs by patients with peripheral artery disease was associated with reduced mortality and better long-term clinical outcomes, particularly for those with moderately increased walking ability.

### Factors influencing perception and participation in home-based exercise

**Fig. 9:**
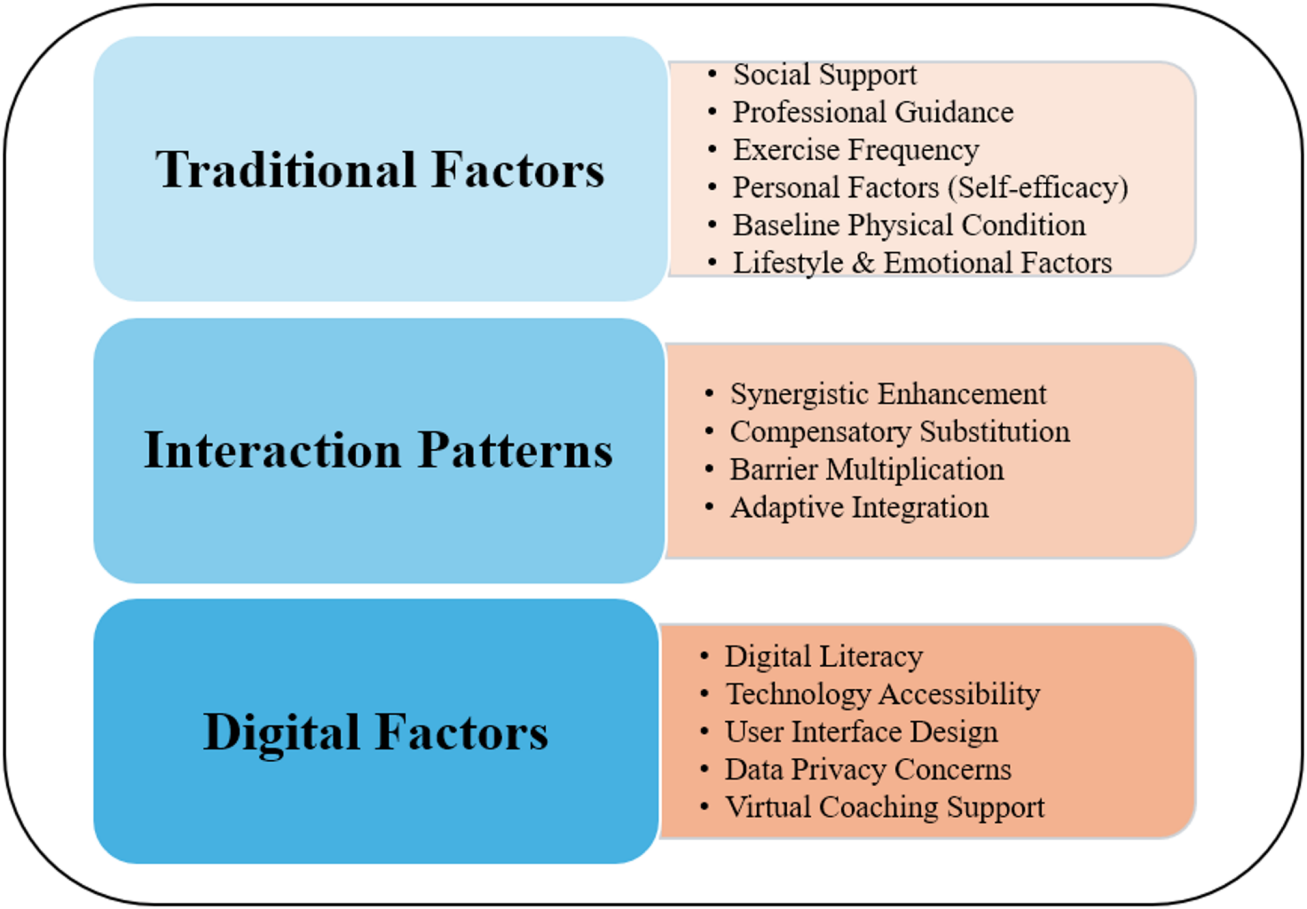
The dual-pathway Model illustrates the dynamic interactions between traditional and digital factors influencing HBE adoption

The main factors influencing the perception and participation in HBE involve various aspects such as social support, professional guidance, exercise frequency settings, personal traits, physical condition, lifestyle habits, and emotional state [14, 51]; Ortega-Pérez de Villar et al., 2020). The factors influencing HBE perception and participation can be conceptualized through a dual-pathway model (Fig. 9) that illustrates the dynamic interactions between traditional and digital factors. This model demonstrates how traditional factors (social support, professional guidance, exercise frequency, personal traits, physical condition, and lifestyle factors) serve as foundational elements that are either enhanced or challenged by digital factors (digital literacy, technology accessibility, user interface design, data privacy concerns, and virtual coaching support). The model reveals four key interaction patterns: (1) Synergistic enhancement, where digital technologies amplify traditional support mechanisms; (2) Compensatory substitution, where digital solutions address gaps in traditional support; (3) Barrier multiplication, where digital challenges compound traditional obstacles; and (4) Adaptive integration, where users develop hybrid approaches combining both traditional and digital elements. This framework provides a more nuanced understanding of how modern HBE programs must address both conventional rehabilitation principles and emerging digital health considerations.

From a traditional perspective, first, social support is an important influential factor. Research has found that emotional encouragement and practical assistance from family members, friends, and peers can significantly improve patients’ adherence to HBE [35]. Social support provides patients with continuous positive stimulation and encouragement, which is a powerful support for sustaining exercise habits.Second, professional guidance plays a crucial role. The guidance, feedback, follow-up, and supplementary educational materials provided by physical therapists and other healthcare professionals can help enhance patients’ exercise motivation, cultivate good exercise habits, and thereby increase the continuous participation in HBE programs [36]. With the development of internet technology and the application of artificial intelligence, online video fitness guidance can also better assist in HBE [82].Third, a reasonable exercise frequency is equally important. Research indicates that for individuals aged 65 and above, if the prescribed exercise frequency is too high (e.g., more than 4 times per week), patient adherence tends to be poorer. In contrast, older adults are more Likely to persist with an exercise frequency of 3 times per week or less [23]. Furthermore, research has found that even for college students, adhering to an exercise frequency of about 3 times per week is more sustainable [70]. Therefore, it is necessary to develop exercise programs with appropriate frequencies. Fourth, personal factors should not be overlooked. These include self-motivation levels, self-efficacy, and past adherence behaviors, all of which can affect the degree of participation in HBE [48]. Additionally, research has found that groups with different levels of self-efficacy exhibit varying levels of participation in HBE [62]. Fifth, baseline physical condition is an influential factor. If an individual has a good level of physical activity or aerobic capacity at baseline, they are more likely to adhere to a HBE program [26]. Conversely, those with poorer physical abilities may have lower sustained participation. Sixth, some lifestyle habits and emotional states are also worth noting. Factors such as lack of time for exercise, forgetfulness, exacerbation of pain during exercise, or negative emotions like depression, anxiety, and helplessness can become obstacles and hindrances [71].

From the perspective of modern digital technology, the technology involved in HBE needs to improve the digital literacy of the user, enabling the researcher or therapist to be able to know how to use the tool. And for the use of these technologies, there is a need for the institution to have the conditions to use the technology to improve the accessibility of the technology. The design of the user interface is also necessary to protect the privacy of the data of the users while using the various online platforms, which is a matter of research ethics. It is commendable that the development of virtual technology can be one of the ways to promote HBE among people, and the scope of technology involved is growing exponentially while continuously exploring its applications, designed in various fields.

### Advantages and prospects of home-based exercise

In recent years, HBE has garnered significant attention in the context of various health conditions and the COVID-19 pandemic. A randomized controlled trial by Tanucan et al. [83] found that HBE effectively improves the health status of adolescents, particularly the cardiorespiratory function of females. For specific populations, Schmitz et al. [75] demonstrated the potential benefits of HBE for breast cancer survivors with lymphedema. During the COVID-19 pandemic, the importance of HBE became even more prominent. Ravalli and Musumeci [69] discussed the physiological benefits of HBE during the pandemic, such as respiratory benefits, while Schwendinger and Pocecco [77] provided evidence-based HBE recommendations to combat physical inactivity; while Newton et al. [52] emphasized the challenges and suggestions for implementing HBE programs for cancer patients. Additionally, Pu et al. [67] studied the influence mechanism of health awareness on HBE, expanding theoretical research in this field.

Technological innovations are bringing more possibilities to HBE. Of course, new technologies such as wearable devices, apps, and VR will bring more assistive tools for HBE, enhancing guidance and enjoyment [15, 79]. Various intelligent sports facilities have effectively encouraged people’s physical activities, and one study suggests that HBE will become an important scenario for the use of intelligent exercise in the future and will promote the spread of HBE [43]. In some special circumstances, HBE will become a necessary and effective form of rehabilitation. Many countries have already included HBE within the scope of health promotion policy support, demonstrating the importance placed on this form of exercise.

As population aging intensifies, the demand for convenient and economical HBE will increase significantly [12]. The advantages and prospects of HBE extend beyond current applications to encompass transformative potential for future healthcare delivery. Our analysis reveals three emerging paradigms that represent significant departures from traditional approaches: (1) Precision HBE, where AI-driven personalization algorithms adapt exercise programs in real-time based on individual physiological responses, behavioral patterns, and environmental factors; (2) Integrated Care Ecosystems, where HBE platforms seamlessly connect with electronic health records, wearable devices, and healthcare provider systems to create comprehensive health management networks; and (3) Predictive Health Maintenance, where continuous monitoring and machine learning algorithms enable proactive intervention before health deterioration occurs. These paradigms suggest that future HBE will transition from reactive treatment tools to proactive health optimization platforms. Our temporal analysis indicates that the field is approaching a critical inflection point where technological convergence will enable unprecedented levels of personalization, accessibility, and effectiveness. The identification of emerging research hotspots such as ‘digital monitoring of home exercise’ and ‘chemotherapy side effects mitigation’ provides clear roadmaps for future investigation and development efforts.

## Conclusion

This study systematically explored the current status of the specific use of digital technologies for home-based exercise in health education. Key research hotspots identified include smart exercise training and remote rehabilitation guidance, artificial intelligence-driven fall risk prevention, wearables and digital musculoskeletal disease interventions, virtual reality technology for neurological disease rehabilitation, mHealth solutions for psychosocial and cardiovascular health testing, rehabilitation tracking systems for cancer patients, and remote web-based diagnosis and rehabilitation of peripheral vascular disease. The United States, Australia, Canada, and the United Kingdom have some of the most active and influential research organizations in this field, reflecting the high priority these countries place on health education in public health. Emerging hotspots such as “digital monitoring of home exercise” and “mitigating chemotherapy side effects” were identified through keyword clustering and burst detection, pointing the way to future developments in the field. Key factors affecting awareness of and participation in home physical activity were considered from two perspectives influencing digital home exercise: social support, professional guidance, exercise prescription, self-efficacy, and environmental factors, and digital literacy, technological accessibility, user interface design, data privacy issues, and virtual tutoring support.

The practical implications of our findings extend across multiple stakeholder groups, providing actionable insights that translate research into real-world applications. For healthcare providers, our dual-factor model offers a systematic approach to assess patient readiness for technology-enhanced HBE programs, while our technology taxonomy provides evidence-based guidance for selecting appropriate digital tools for specific patient populations and conditions. For technology developers, our temporal analysis reveals market opportunities in underexplored areas such as neurological rehabilitation gaming and AI-powered fall prevention systems. For policy makers, our findings support the development of digital health literacy programs and infrastructure investments that address identified barriers to HBE adoption. Most significantly, our research provides a theoretical foundation for developing next-generation HBE interventions that combine the most effective elements of traditional rehabilitation approaches with cutting-edge digital technologies, ultimately improving health outcomes while reducing healthcare costs and increasing accessibility for diverse populations.

While this study provides comprehensive insights into HBE research trends and technology applications, several limitations should be acknowledged, which simultaneously point toward future research opportunities. First, our analysis focused primarily on English-language publications in the WOS database, potentially missing important contributions from other linguistic and cultural contexts. Future research should incorporate multilingual databases and cross-cultural validation studies to ensure global applicability of our findings. Second, the rapid pace of technological advancement means that emerging technologies such as augmented reality, blockchain-based health records, and advanced AI algorithms may not be fully captured in our analysis. Longitudinal studies tracking technology adoption patterns and effectiveness outcomes will be essential for maintaining current understanding of this dynamic field. Third, while our dual-factor model provides a theoretical framework for understanding user adoption, empirical validation through large-scale intervention studies is needed to confirm the predictive value of our proposed relationships.

## Data Availability

No datasets were generated or analysed during the current study.
